# Supplementary material for: Ferrocene-Doped Polystyrene Nanoenzyme and DNAzyme Cocatalytic SERS Quantitative Assay of Ultratrace Pb2+
Source: Nanomaterials (Basel). 2022 Apr 7;12(8):1243. doi: 10.3390/nano12081243 (PMC9027246; doi:10.3390/nano12081243)
Supplement: Supplementary file 1 [file nanomaterials-12-01243-s001.zip › nanomaterials-1614063-Sl.pdf]

# Supporting Information

# Ferrocene-Doped Polystyrene Nanoenzyme and DNAzyme Cocatalytic SERS Quantitative Assay of Ultratrace $Pb^{2+}$

**Chongning Li [1,2], Zhenghong Wang [1,2] and Zhiliang Jiang [2,*]**

1 School of Public Health, Guilin Medical University, Guilin 541199, China; lcn7882342@163.com (C.L.); zh27685@163.com (Z.W.)
2 Guangxi Key Laboratory of Environmental Pollution Control Theory and Technology, Guilin 541006, China
* Correspondence: zljiang@mailbox.gxnu.edu.cn

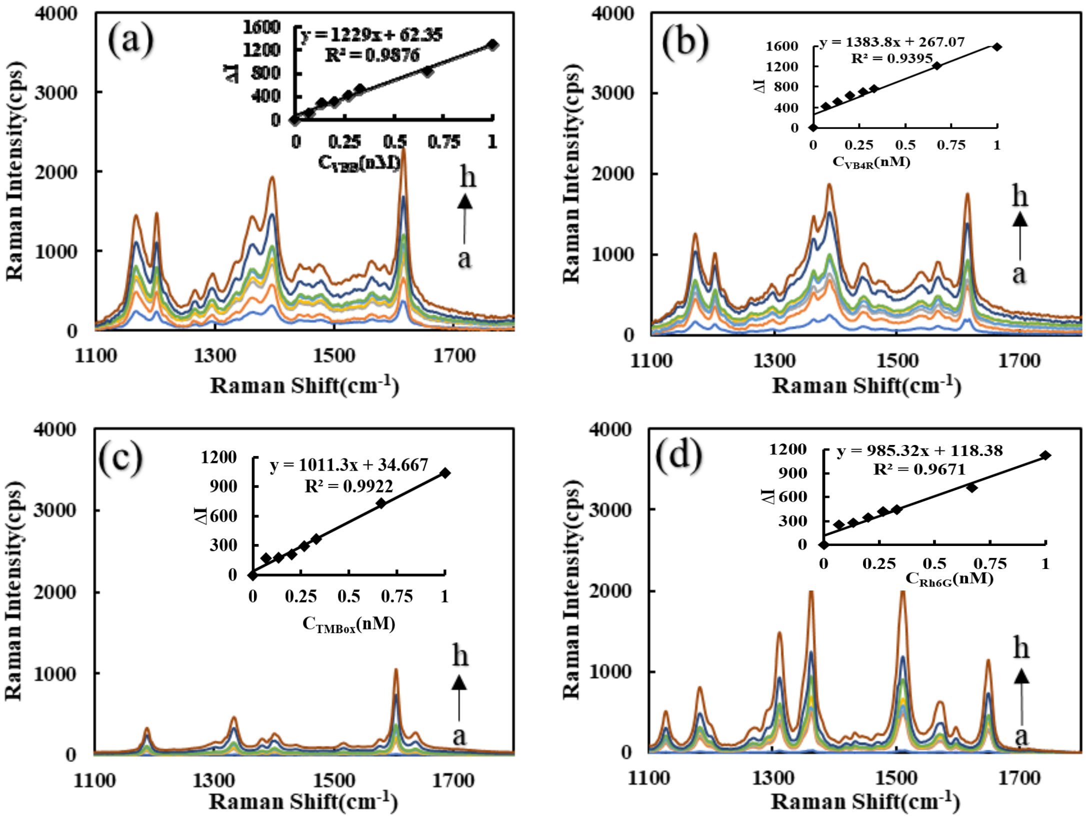

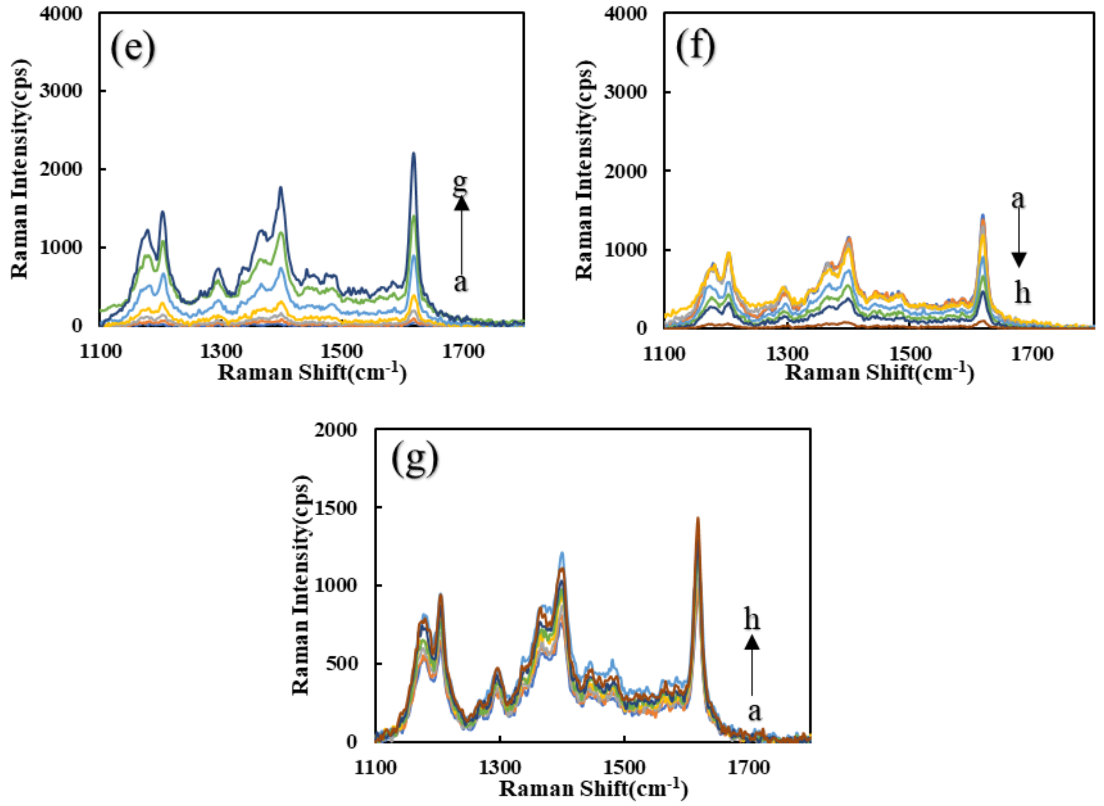


**Figure S1.** SERS spectra of nanocatalytic analysis system with different molecular probes.
(a) a to h: (0, 0.067, 0.13, 0.2, 0.27, 0.33, 0.67, 1) nM $Pb^{2+}$＋HM+Apt+$PN_3$+$AgNO_3$+SF+0.33 μM VBB; (b) a to h: (0, 0.067, 0.13, 0.2, 0.27, 0.33, 0.67, 1) nM $Pb^{2+}$+HM+Apt+$PN_3$+$AgNO_3$+SF+0.33 μM VB4R; (c) a to h: (0, 0.067, 0.13, 0.2, 0.27, 0.33, 0.67, 1) nM $Pb^{2+}$+HM+Apt+$PN_3$+$AgNO_3$+SF+0.33 μM $TMB_{OX}$; (d) a to h: ( 0, 0.067, 0.13, 0.2, 0.27, 0.33, 0.67, 1) nM $Pb^{2+}$+HM+Apt+$PN_3$+$AgNO_3$+SF+0.33 μM Rh6G; (e) a to g: (0, 0.5, 1.0, 2, 5, 10, 20) mg/L $PN_3$+$AgNO_3$+SF+VBB; (f) a to h: (0, 0.05, 0.1, 0.2, 0.5, 1, 2, 10) nM Apt+$PN_3$+$AgNO_3$+SF+VBB; (g) a to h: (0, 0.67, 2, 4.67, 6, 7.3, 8.67, 10) nM HM+$Pb^{2+}$+Apt+$PN_3$+$AgNO_3$+SF+VBB.

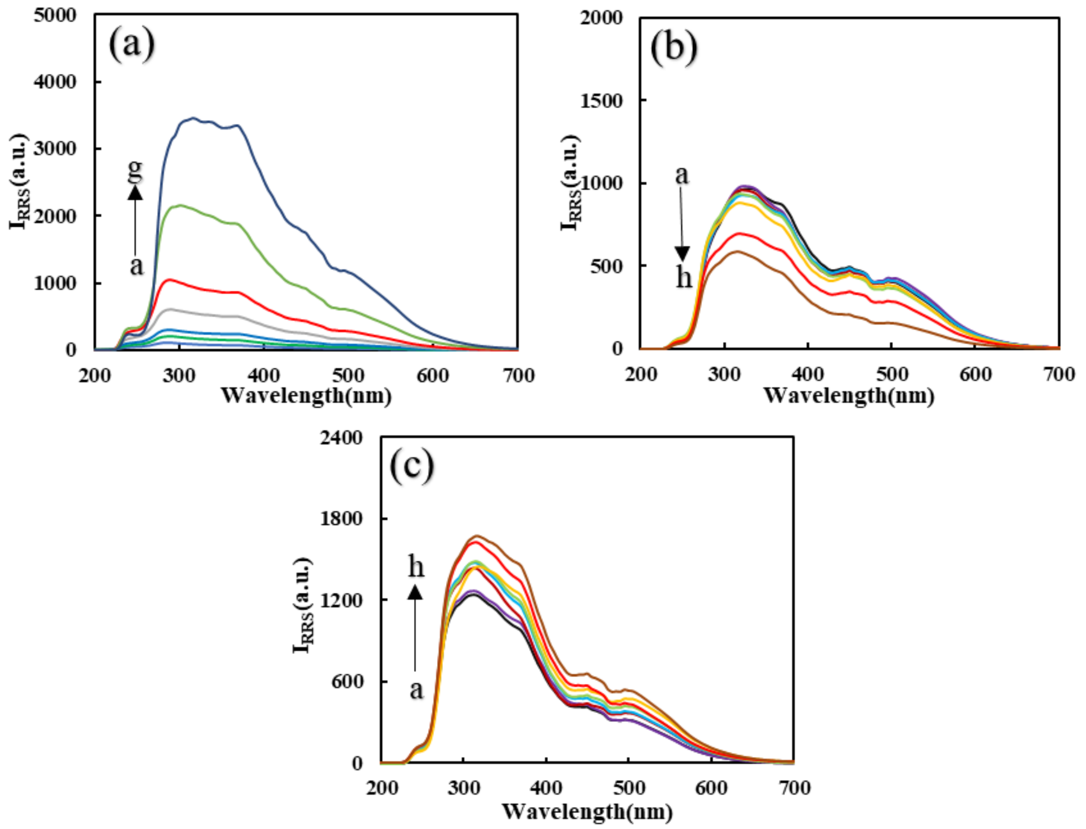


**Figure S2.** RRS spectra of nanocatalysis and analysis system.
(a) a to g: (0, 0.5, 1, 2, 5, 10, 20) mg/L $PN_3$+$AgNO_3$+SF; (b) a to h: (0, 0.05, 0.1, 0.2, 0.5, 1, 2, 10) nM Apt+$PN_3$+$AgNO_3$+SF; (c) a to h: (0, 0.67, 2, 4.67, 6, 7.3, 8.67, 10) nM HM+$Pb^{2+}$+Apt+ $PN_3$+$AgNO_3$+SF.

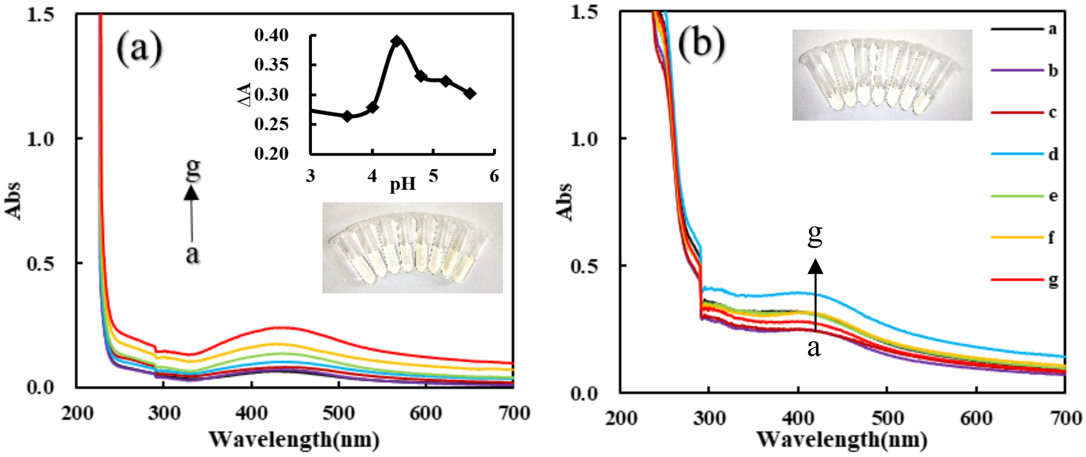

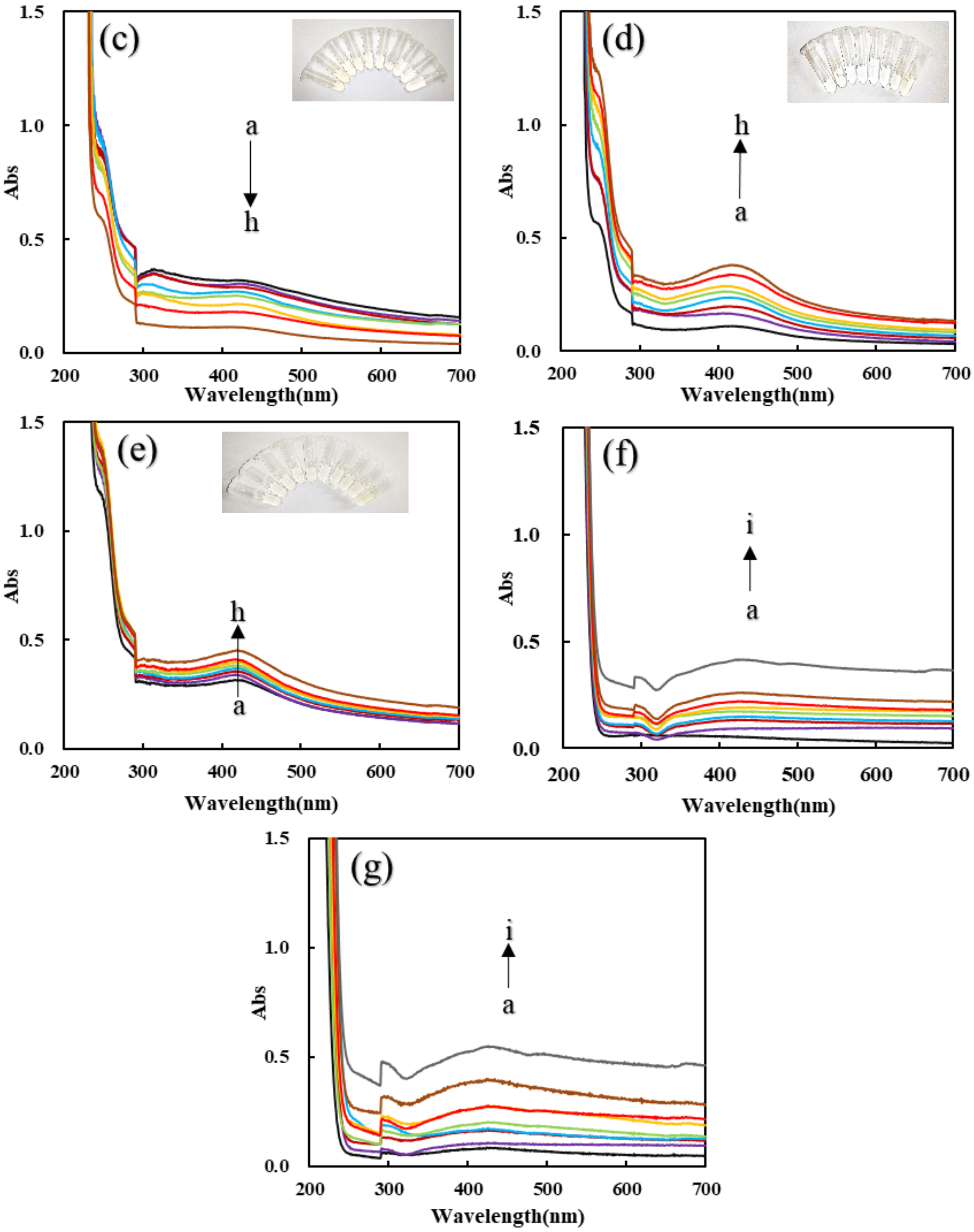


**Figure S3.** UV absorption spectra of nanocatalytic and analysis system.
(a) a to g: (0, 0.5, 1.0, 2, 5, 10, 20) mg/L $PN_3$+$AgNO_3$+SF; (b) a to g: (0, pH=3.6, 4 , 4.4, 4.8, 5.2, 5.6) +Apt+$PN_3$+$AgNO_3$+SF; (c) a to h: (0, 0.05, 0.1, 0.2, 0.5 , 1, 2, 10) nM Apt+$PN_3$+ NaAc-HAc+ $AgNO_3$+SF; (d) a to h: (0, 0.67, 2, 4.67 , 6, 7.3, 8.67, 10) nM HM+$Pb^{2+}$+Apt+$PN_3$+$AgNO_3$+SF; (e) a to h: (0, 0.067, 0.13, 0.2, 0.27, 0.33, 0.67, 1) nM $Pb^{2+}$+ HM+ Apt+ $PN_3$+$AgNO_3$+SF. (f) a to i: (0, 0.05, 0.1, 0.2, 0.3, 0.4, 0.5 ,1,2) nM $Pb^{2+}$+$PN_3$+Apt+HM+$AgNO_3$+SF; (g) a to i: (0 , 0.05, 0.1, 0.2, 0.3, 0.4, 0.5, 1, 2) nM $Pb^{2+}$+$PN_{Fer3}$+Apt+HM+$AgNO_3$+SF.

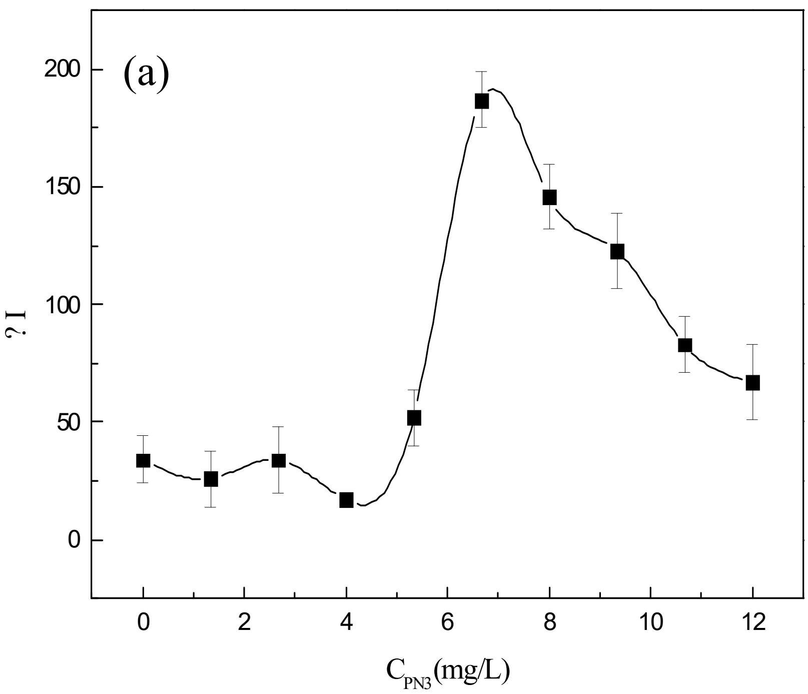
(a)
? I
200
150
100
50
0
0
2
4
6
8
10
12
$C_{PN3}$(mg/L)

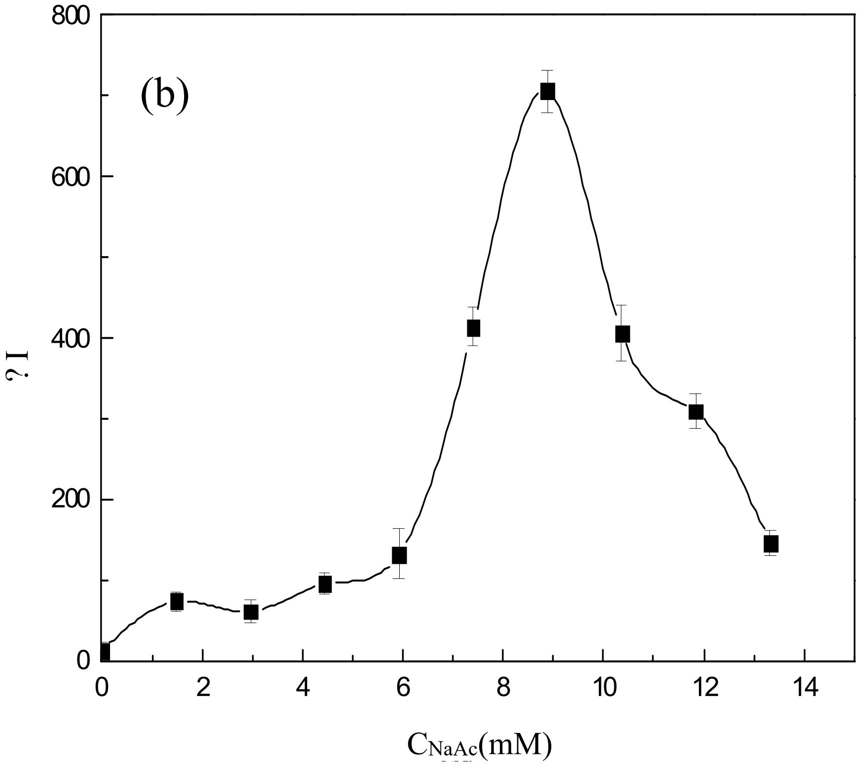
(b)
? I
800
600
400
200
0
0
2
4
6
8
10
12
14
$C_{NaAc}$(mM)

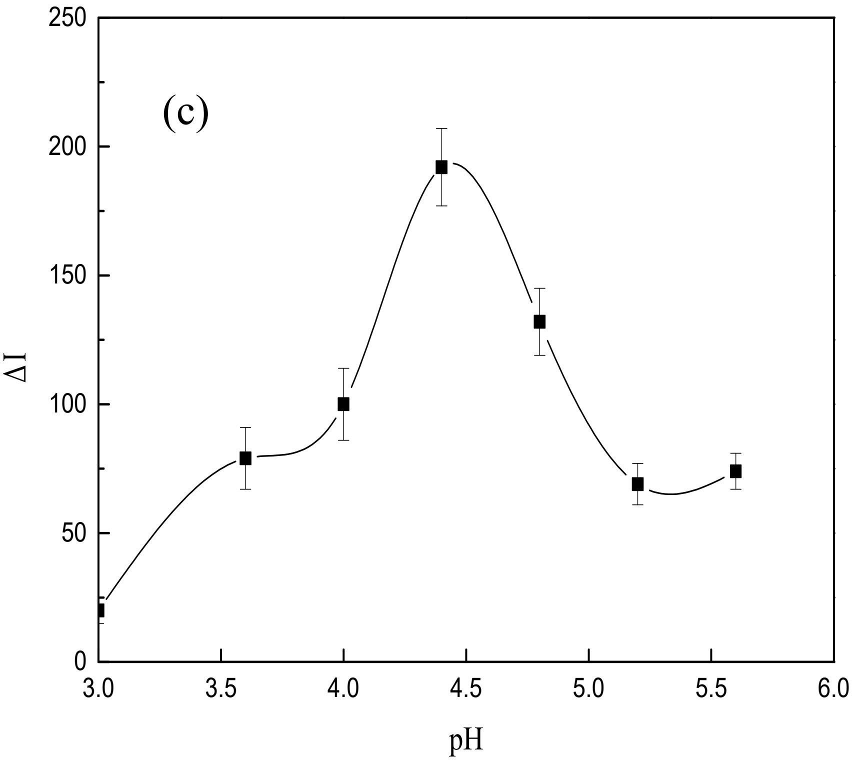
(c)
ΔI
pH
250
200
150
100
50
0
3.0
3.5
4.0
4.5
5.0
5.5
6.0

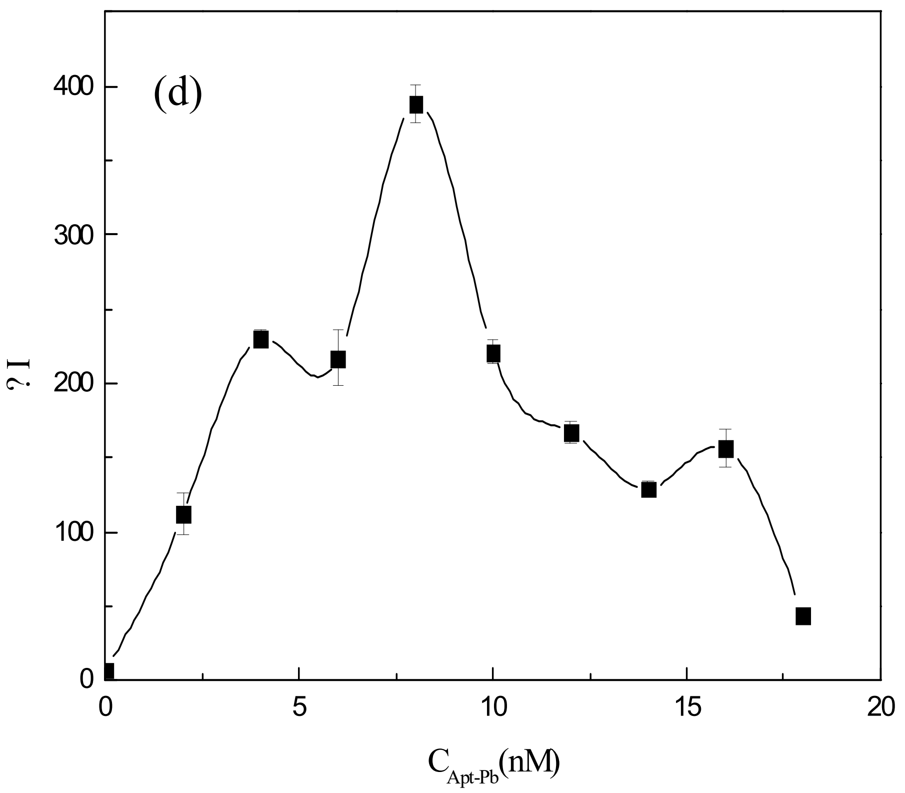
(d)
?I
C Apt-Pb (nM)
400
300
200
100
0
0
5
10
15
20

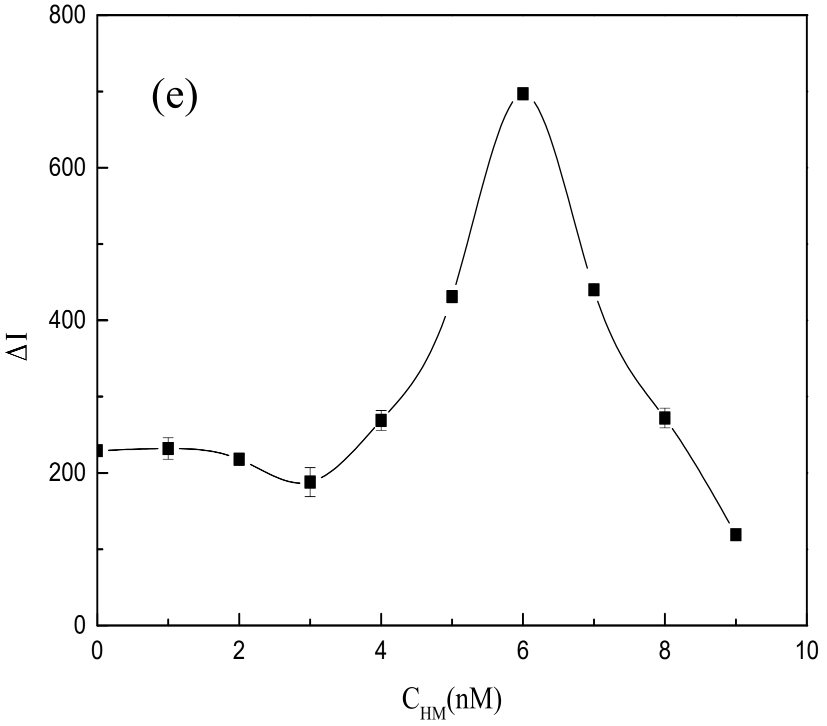
(e)
ΔI
C_HM(nM)
0
200
400
600
800
0
2
4
6
8
10

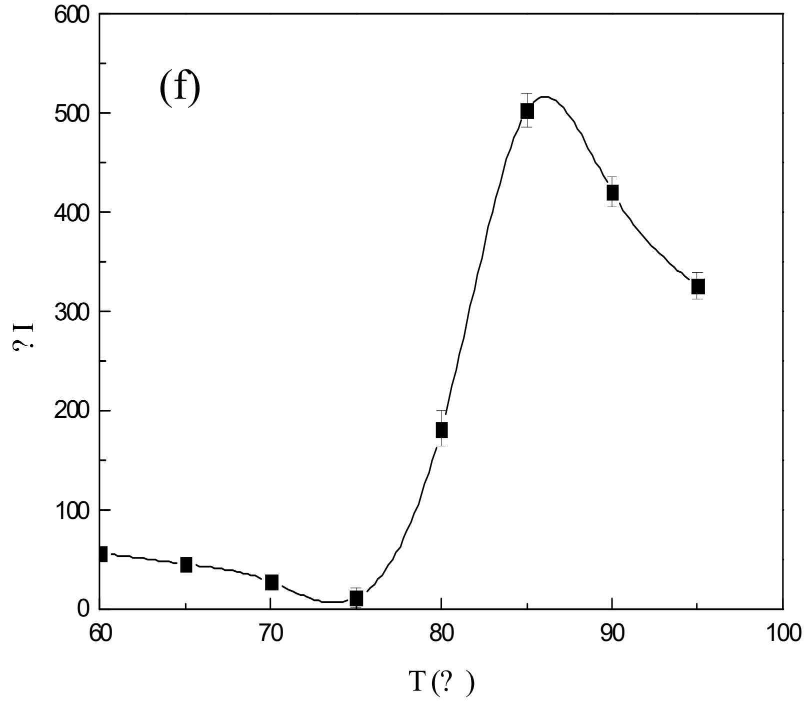
(f)
? I
T (? )
0
100
200
300
400
500
600
60
70
80
90
100

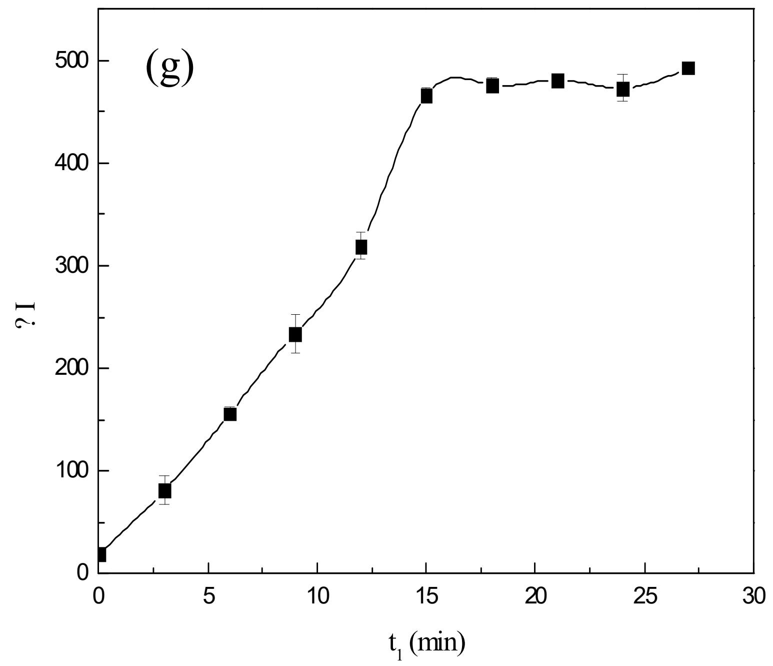


**Figure S4.** Optimization of reaction conditions.
(a) The influence of the concentration of $PN_{Fer3}$; (b) NaAc concentration; (c) The effect of pH on buffer system; (d) The effect of Apt concentration; (e) The effect of HM concentration; (f) The influence of reaction temperature; (g) The influence of reaction time.

**Table S1.** Comparison of the nanocatalysis of $AgNO_3$-SF reaction by means of the slope.

| Method | Catalyst | Linear range | Linear equation | Coefficient |
|---|---|---|---|---|
| RRS | $PN_1$ | 0.5-20 mg/L | $\Delta I = 93.7C + 28.5$ | 0.9994 |
| | $PN_2$ | 0.5-20 mg/L | $\Delta I = 106.4C + 47.3$ | 0.9977 |
| | $PN_3$ | 0.5-20 mg/L | $\Delta I = 166.0C + 83.2$ | 0.9957 |
| | $PN_4$ | 0.5-20 mg/L | $\Delta I = 91.6C + 183.3$ | 0.9712 |
| | $PN_{Fer1}$ | 0.5-20 mg/L | $\Delta I = 198.3C + 6.2$ | 0.995 |
| | $PN_{Fer2}$ | 0.5-20 mg/L | $\Delta I = 208.7C + 30.7$ | 0.9978 |
| | $PN_{Fer3}$ | 0.5-20 mg/L | $\Delta I = 291.3C + 161.9$ | 0.9787 |
| | Fer | 0.11-1.08 mM | $\Delta I = 63.047C - 11.4$ | 0.4002 |
| Abs | $PN_1$ | 0.5-20 mg/L | $\Delta A = 0.011C + 0.07$ | 0.7879 |
| | $PN_2$ | 0.5-20 mg/L | $\Delta A = 0.012C + 0.13$ | 0.7880 |
| | $PN_3$ | 0.5-20 mg/L | $\Delta A = 0.0097C + 0.06$ | 0.8414 |
| | $PN_4$ | 0.5-20 mg/L | $\Delta A = 0.014C + 0.07$ | 0.8515 |
| | $PN_{Fer1}$ | 0.5-20 mg/L | $\Delta A = 0.014C + 0.02$ | 0.9469 |
| | $PN_{Fer2}$ | 0.5-20 mg/L | $\Delta A = 0.013C + 0.04$ | 0.8992 |
| | $PN_{Fer3}$ | 0.5-20 mg/L | $\Delta A = 0.015C + 0.01$ | 0.9818 |
| | Fer | 0.11-1.08 mM | $\Delta A = 0.067C + 0.03$ | 0.3354 |

| | | | | |
|---|---|---|---|---|
| SERS | $PN_1$ | 0.5-20 mg/L | $\Delta I = 60.4C + 13.6$ | 0.9983 |
| | $PN_2$ | 0.5-20 mg/L | $\Delta I = 76.6C + 89.1$ | 0.9775 |
| | $PN_3$ | 0.5-20 mg/L | $\Delta I = 109.7C + 125.5$ | 0.9714 |
| | $PN_4$ | 0.5-20 mg/L | $\Delta I = 106.5C + 301.2$ | 0.8971 |
| | $PN_{Fer1}$ | 0.5-20 mg/L | $\Delta I = 116.3C + 215.7$ | 0.9657 |
| | $PN_{Fer2}$ | 0.5-20 mg/L | $\Delta I = 136.1C + 199.4$ | 0.9619 |
| | $PN_{Fer3}$ | 0.5-20 mg/L | $\Delta I = 236.6C - 7.1$ | 0.9985 |
| | Fer | 0.11-1.08 mM | $\Delta I = 9.81C + 13.8$ | 0.0837 |

**Table S2.** Comparison of methods reported to detect $Pb^{2+}$.

| Method | principle | Linear range | DL | Features | Ref. |
|---|---|---|---|---|---|
| ECL | The signal increases when $Pb^{2+}$ is combined with Apt. | 0.1-50 nM | 0.05 nM | Sensitive, but the sensor preparation is complicated. | [30] |
| FL | AuNPs inhibit the fluorescence of graphene. When $Pb^{2+}$ is present, AuNPs-graphene will be separated and the signal will be restored. | 50-1000 nM | 10 nM | Selective, but sensitivity is low. | [31] |
| FL | The functional groups on the surface of fluorescent nanoparticles bind to $Pb^{2+}$ to inhibit fluorescence. | 50-900 nM | 26 nM | high recovery, high-through put, and fast. | [32] |
| ECL | The combination of $Pb^{2+}$ and Apt in the detection electrode causes a signal change. | 0.1-1000 ng/mL | 0.03 ng/mL | Good reproducibilit y and high selectivity. | [33] |
| Hydrogel Capillary Sensor (DHCS) | In the presence of $Pb^{2+}$, the substrate chain breaks, causing the hydrogel to partially break, and the pore size of the hydrogel membrane blocking the capillary end changes. | 0.01-50 μM | 10 nM | Miniature, portable, fast measurement, but insufficient accuracy. | [34] |
| Abs | The addition of $Pb^{2+}$ ions will produce complexes and produce a color change visible to the naked eye. | -- | 40 nM | Good selectivity and reversible color. | [35] |
| FIOES | Magnetic cobalt nanoparticles are used as adsorbent materials to enrich $Pb^{2+}$, and then analyzed. | -- | 4 μg/L | High sensitivity, continuous detection | [36] |
| DLS | The aggregation of $Pb^{2+}$ and GSH-gold nanoparticles to form a chelate complex causes the DLS signal to | -- | 100 ppt | Fast on-site inspection. | [37] |

| | | | | | |
|---|---|---|---|---|---|
| SERS | increase sharply with the increase of the particle size. When $Pb^{2+}$ binds to the substrate, a certain amount of AuNPs conjugates will detach from the gold surface, causing the Raman signal to weaken | -- | 20 nM | Sensitive and accurate. | [38] |
| SERS | $Pb^{2+}$, HM and Apt combine to form DNase and nanoenzyme to co-amplify the signal. | 0.05-2 nM | 0.03 nM | Hihgly sensitive. | This method |

* ECL-electric chemiluminesence, FL- Fluorescence, FIOES-flow injection inductively coupled plasma optical emission spectrometry, DLS- dynamic light scattering.

**Table S3.** The influence of interfering substances on the determination of $Pb^{2+}$.

| Interfering substances | Times | Relative error (%) | Interfering substances | Times | Relative error (%) |
|---|---|---|---|---|---|
| $K^{+}$ | 500 | -0.8 | $HCO_3^{2-}$ | 500 | -6.6 |
| $NO_2^{-}$ | 500 | -8.1 | $P_2O_7^{4-}$ | 500 | -5.4 |
| $Co^{2+}$ | 200 | -8.2 | $Cr^{3+}$ | 100 | -0.12 |
| $Ca^{2+}$ | 500 | 4.7 | $Cu^{2+}$ | 500 | -8.8 |
| $Zn^{2+}$ | 200 | 8.5 | $Hg^{2+}$ | 500 | -8.5 |
| $Mg^{2+}$ | 200 | 6.3 | $Na^{+}$ | 500 | 3.5 |
| $Al^{3+}$ | 500 | -2.7 | $Fe^{2+}$ | 200 | 7.5 |
| $Fe^{3+}$ | 500 | -4.1 | $NH_4^{+}$ | 100 | 7.6 |
| $HPO_4^{2-}$ | 200 | 4.0 | $Ac^{-}$ | 500 | -2.9 |
| $H_2PO^{4-}$ | 500 | -5.9 | $NO_3^{-}$ | 500 | -8.3 |
